# Cognitive Assessment of Japanese Older Adults with Text Data Augmentation

**DOI:** 10.3390/healthcare10102051

**Published:** 2022-10-17

**Authors:** Toshiharu Igarashi, Misato Nihei

**Affiliations:** 1 Department of Human and Engineered Environmental Studies, The University of Tokyo, Kashiwanoha 5-1-5, Chiba 277-8563, Japan; 2 Institute of Gerontology, The University of Tokyo, 3-1, Hongo 7-chome, Bunkyo-ku, Tokyo 113-8654, Japan

**Keywords:** healthcare, assistive technologies, natural language processing, cognitive function, data augmentation

## Abstract

Current medical science has not yet found a cure for dementia. The most important measures to combat dementia are to detect the tendency toward cognitive decline as early as possible and to intervene at an early stage. For this reason, screening for dementia based on language ability has attracted much attention in recent years. However, in most of the previous studies, the cohort of people with dementia has been smaller than the control cohort. In this paper, we use a pre-trained Japanese language model for text analysis and evaluate the effectiveness of text augmentation on a dataset consisting of Japanese-speaking healthy older adults and those with mild cognitive impairment (MCI). We also examined what tasks contributed to the results. This experimental setting can also be used to detect other diseases that may affect the language areas of the brain outside of the hospital.

## 1. Introduction

Dementia is “a chronic decline or loss of various cognitive functions resulting in the inability to lead a normal daily and social life” and is an acquired disorder of intelligence [1]. Cognitive functions are essential for planning and carrying out daily activities such as cleaning, washing, eating, and going out [2]. Therefore, people with dementia may not be able to carry out their daily schedules and activities, which may cause serious challenges [3]. As of 2020, 50 million people worldwide have Alzheimer’s disease, and this number nearly doubles every 20 years, increasing to 152 million by 2050 [4,5]. However, during the past two decades, all clinical trials have failed to find a cure.

Although current medical science has not yet found a cure for dementia, it is possible to slow the progression of dementia with pharmacotherapy and behavioral therapy. Therefore, it is most important to detect the tendency toward cognitive decline as early as possible and to intervene at an early stage, just as in the case of cancer. Positron emission tomography (PET) and magnetic resonance imaging (MRI) are commonly used to test for dementia, and they are not only time-consuming but also require the medical facilities to use expensive testing equipment [6]. Other methods, such as Mini-Mental State Examination (MMSE) and Hasegawa Dementia Scale (HDS-R), are quick and easy screening methods, but since the questions on the test form are fixed, people might remember questions if they are tested regularly [7,8]. Therefore, the questions are unsuitable for periodic monitoring. In addition, the fact that cognitive function is being tested places a mental burden on the test subjects. In fact, many older people refuse to be tested for dementia, and it has been reported that 16% of people with Alzheimer’s disease show catastrophic reactions such as anxiety, anger, and refusal during cognitive testing [9].

Therefore, screening for dementia based on the verbal abilities of older adults has recently attracted attention. Such screening is not physically invasive and does not require long periods of time in a medical facility. In addition, it is possible to monitor cognitive function on a regular basis, and thus, it may be possible to detect the transition of cognitive function over time. Therefore, the purpose of this study is to verify whether text augmentation works effectively on a dataset of healthy older adults and those with mild cognitive impairment (MCI). We also examine which task contributes to the classification results for the 10 episodic, picture description, and animation description tasks in the dataset.

## 2. Related Works

In general, people with dementia are known to have reduced language ability compared to healthy control subjects. Many studies have been conducted to screen for dementia by language ability. Studies focusing on language began with the Nun Study in 1996 [10], and the number of studies using machine learning to differentiate people with dementia from healthy control subjects has been gradually increasing [11,12].

According to previous systematic reviews [13,14], machine learning-based assessments of cognitive function can be classified into three main categories, depending on the type of features employed: (i) linguistic features, (ii) acoustic features, and (iii) other types of features such as expressive features or features that depict specific shapes. Among them, this study focuses on linguistic features that can deal with fillers and feature words in classification tasks.

### 2.1. Linguistic Features

Linguistic features can be extracted by employing three types of analyses, i.e., (1) the primary lexical level analysis, (2) a semantic analysis, and (3) a sentence-level syntactic analysis.

(1) For the primary lexical level analysis, an automated primary lexical analysis, that is, a lexical or word level analysis, can generate objective language metrics and provide valuable insight into cognitive function. In its most basic approach, the body of a text is treated as a bag of words. This means that the order of words in the text is not considered.

Jarrold et al. used speech data from both healthy people (n=23) and people with dementia (n=22) to extract the number of parts of speech, the semantic density, and the word industry categories using a linguistic inquiry and word count (LIWC), a tool for categorizing word industries, and used them as features for machine learning [11]. In addition, Asgari et al. reported that speech data (daily chat) obtained from participants with mild cognitive impairment (n=14) and healthy participants (n=21) can be used to classify healthy control (HC) and MCI with up to 73% accuracy using LIWC and reported that LIWC can differentiate between HC and MCI with an accuracy of up to 84% [15]. Fraser et al. used several primary lexical features in their analysis of Alzheimer’s disease (AD) participants [16]. The authors used the type token ratio (TTR) as a measure of lexical diversity or richness to differentiate between healthy older control participants (n=16) and successfully identified a small sample of AD participants (n=8). In addition, they considered the usage of other parts of speech (nouns, pronouns, adjectives, and verbs). In particular, the TTR and adjective rates all showed strong group differences between participants with AD and healthy control subjects; the groups were classified with 87.5% cross-validation accuracy.

(2) For the semantic analysis, the semantic similarity of natural language is usually measured computationally by embedding the text into a high-dimensional vector space that represents its semantic content. The notion of distance between vectors can then be used to quantify the semantic similarities and differences between words and sentences represented by vector embedding.

Snowdon et al. calculated the semantic density (the number of propositions in a sentence divided by the number of words) and grammatical complexity from autobiographies written by 93 nuns when they were in their 20 s. They showed that a lower semantic density and grammatical complexity during youth were associated with a lower cognitive function in later life and reported a relationship between these values and cognitive function. Kemper et al. also reported that the grammatical complexity decreased with age regardless of the presence or absence of dementia, but semantic density decreased only in the dementia group [17].

(3) A sentence-level syntactic analysis can also provide important insight into cognitive functioning from the order of words in a sentence and sentences in a paragraph. To produce free-flowing speech, we need to determine not only which words best convey an idea, but also the order in which the words form a sentence. The complexity of the sentences we construct provides insight into cognitive linguistic health. In this section, we provide an overview of the various methods used to measure syntactic complexity as a proxy for cognitive health. There are many common structural measures of language that are easy to calculate, such as the average length of a clause, the average length of a sentence, and the ratio of the number of clauses to the number of sentences.

Orimayre et al. extracted several syntactic and lexical features from a corpus consisting of people with dementia (n=314) and healthy people (n=242) provided by the Dementia Bank, and classified them by machine learning, achieving an F-measure of 0.74 [18]. Fraser et al. conducted a picture description task on participants with mild cognitive impairment (n=37) and healthy control subjects (n=37). Language features were extracted from the obtained speech data and differentiated, resulting in an AUC value of up to 0.87 [18]. Methods using deep learning have also recently been proposed, and Klekar et al. reported that they achieved 91% accuracy in classifying people with dementia and healthy people using the Dementia Bank [19]. A review paper also investigated the content of a paper experimenting with an image description task in a corpus [20]. Currently, as GPU performance has improved, it has become easier to construct models that are computationally expensive, and high accuracy can be expected from sentence-level parsing.

### 2.2. Dealing with Imbalanced Data

Collecting a large amount of data from people with dementia is not easy because it can be difficult to obtain their consent due to research ethics issues. As a result, there are imbalanced data based on a large amount of healthy older adults and a small number of cognitively impaired older adults. In some cases, it is difficult to know whether the classification accuracy reported in the previous studies is correct (in the imbalanced data with 90% of healthy older adults, if they always presume that the subjects are healthy, the accuracy would be 90%). Furthermore, the sample sizes are so different that we do not know which method or task contributes to the classification results.

Under-sampling is the simplest method to deal with imbalanced data, but it leaves the problem that the total amount of data becomes small [21]. One method of oversampling that adjusts minority data to the majority is to balance the data by augmentation. However, data augmentation is often used with image data, and its application to text data is not common [22]. In addition, it is not known whether text augmentation works as effectively with characteristic text data of people with dementia as it does with normal text. Data augmentation is a means of dealing with imbalanced data. Particularly successful in the field of imaging, this method bulks up similar data by inverting, scaling, or otherwise altering the image in various ways. This is said to have the advantage of improving generalization performance and classification accuracy on small datasets.

However, data augmentation is not very common in natural language processing, and no established method has yet been found. Jason Wei et al. proposed easy data augmentation (EDA), a method to increase the number of similar texts, to see its effect on classification accuracy on small datasets, Stanford Sentiment Treebank, and other datasets [23]. This study suggests that EDA may be able to achieve the same level of accuracy as conventional methods even when the dataset size is less than half of that required by conventional methods. It is reported that EDA alone can reproduce accuracy with only 500–1000 datasets.

In addition, a list of stopwords is required for text augmentation. Because some sentences may contain words that are meaningless when amplified, these words should be excluded from text augmentation as stopwords. For Japanese stopwords, “Slothlib” proposed by Oshima et al. [24]. Slothlib is a list of words separated by “ ”, which excludes the list of words contained within it from text augmentation. However, because people with dementia are known to use pronouns more frequently, Slothlib, which includes the words, cannot be used [25].

## 3. Method

### 3.1. Dataset

#### 3.1.1. Dataset Overview

We evaluated the proposed classification model using a corpus of older adults with control groups created by Shihata et al. [25]. This corpus contains speech data from 60 individuals (30 males and 30 females) and older adults who performed three types of stimulus tasks, with accompanying MMSE results as an assessment of cognitive function. The MMSE results were obtained by trained clinical psychologists and others.

The three types of stimulus tasks included an episodic task (Table 1), a picture explanatory task for a Cookie Theft Picture [26], and an animation explanatory task for animation of NAIST DOGS produced by the Nara Institute of Science and Technology (NAIST). The episodic task includes 10 tasks: (1a) recent sad event, (1b) when did it happen, (2a) recent event that made you feel anxious, (3a) recent event that made you angry, (4a) recent event that made you feel disgusted, (5a) recent surprising event, (6a) recent pleasant event, (6b) when did it happen, (7a) people you admire, (8a) what you are currently passionate about. In addition, one image and animation task gave a total of 12 tasks. Subjects were instructed to speak freely for one to two minutes in response to each task question, and their utterances were recorded as audio and accompanied by manually transcribed text data.

**Figure 1:**
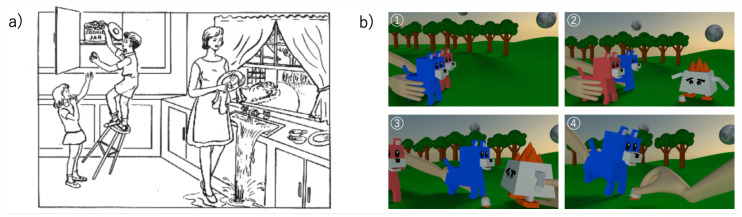
(**a**) Images used in picture description tasks (copyright Goodglass). (**b**) Movie used in the animation description task (public domain).

In the picture description task, the subjects were shown the cookie theft picture by Goodgiass et al. widely used in previous studies, and were asked to talk about the contents of the picture (Figure 1). In the animation description task (ADT), each subject watched the NAIST dog story, a computer animation whose copyright has been waived, and was asked to give an explanation of what happened within one to two minutes (if the subject had little to say, less than 1 minute was acceptable). In the movie, (1) a blue dog and a red dog are taking a walk, (2) the dogs come across a large unidentified creature tormenting a small unidentified creature, (3) the blue dog surprises the large unidentified creature, and (4) the small unidentified creature thanks the dogs for their help, and leaves on the back of the blue dog.

#### 3.1.2. Cognitive Function of the Subject in the Dataset

The MMSE is one of the most commonly used screening methods for detecting dementia. The MMSE is a 30-point cognitive function test comprising 11 items: time and place recognition, immediate and delayed word play, calculations, object calling, sentence recitation, three-step verbal commands, writing commands, writing, and graphic copying. In the MMSE, 23 points or less indicates potential dementia (sensitivity of 81%; specificity of 89%), and 27 points or less indicates potential MCI (sensitivity of 45–60% and specificity of 65–90%) [27,28,29,30].

In this study, older people with an MMSE score of 28 points or more were considered as healthy older people; older people with an MMSE score of 23 points or more and 27 points or less were considered to have MCI. Based on the results of the MMSE, out of 60 older people, 15 were judged to have MCI, and 45 were judged as healthy older people (Table 2).

### 3.2. Classification Method

Generalized language models that have been pre-trained on large corpus perform well on natural language tasks. While many pre-trained transformers for English have been published, there are few model options available, especially for Japanese texts. In this study, we use BERT (Bidirectional Encoder Representations from Transformers), a pre-trained Japanese language model that is considered a ubiquitous baseline for NLP experiments [31]. BERT is based on Transformer and provides powerful encoding for sentences and text. NEologd was used as the Japanese dictionary, and MeCab was used for tokenization of sentences [32].

### 3.3. Parameters in Fine Tuning

For automating machine learning, automated machine learning (AutoML) is a method frequently proposed in recent papers. The AutoML is a learning model building service provided by Google that allows users to build models using data on the Google Cloud Platform. However, AutoML has a drawback that the trend of model optimization becomes a black box, and it is not suitable for the purpose of examining the possibility of text augmentation of MCI patients, as in this study. Therefore, in this study, multiple parameters are set for fine tuning, and the optimal parameters are searched for.

The optimal parameters values depends on the task, but according to the authors of BERT, the following settings are recommended as parameters that work for common tasks. Batch size: 16, 32. Learning rate: 5 $\times \;10^{-5}$, 3 $\times \;10^{-5}$, 2 $\times \;10^{-5}$. Number of epochs: 2, 3, 4. In this study, an early stopping program was applied to prevent over-fitting, which would be optimized only for the training data and thus lose its generality. The number of learning epochs was fixed at 4, the maximum value, because learning is automatically stopped when it is judged to be sufficiently learned.

The parameters recommended by the authors of BERT are for 128 tokens per sentence, but some of our data with 512 tokens per sentence may be too large. Therefore, we have added candidates as small as 1, 2, 4, and 8 to the recommended batch sizes. In summary, we experimented with model building with all of the following 18 parameter patterns to find the optimal parameters.

– Batch size: 1, 2, 4, 8, 16, 32;
– Learning rate: 5 $\times \;10^{-5}$, 3 $\times \;10^{-5}$, 2 $\times \;10^{-5}$;
– Number of epochs: 4.

### 3.4. Data Cleansing

The dataset contained words for marking the beginning and end of the recording, but these are common to all the data and do not make a difference, so they were removed. The transcribed data that had arbitrary line breaks were also removed. The maximum length of a sentence that can be handled by BERT is 512. There were several sentences in the dataset longer than 512 characters; these were systematically truncated.

### 3.5. Setting the Stop Word

In order to avoid augmentation of meaningless words that are not necessary from the analysis point of view, stopwords were set. While referring to Slothlib by Oshima et al., 288 words were used as stopwords, excluding directives and words that are equivalent to directives.

### 3.6. Easy Data Augmentation (Eda)

We implemented easy data augmentation, a method proposed by Jason Wei et al. to increase the number of similar texts. EDA consists of four algorithms. (1) Synonym replacement, which randomly selects a word in a sentence and replaces it with one of a list of synonyms for that word (excluding stopwords). (2) Random Insertion randomly selects a word in a sentence and randomly inserts it at a different position in the sentence (excluding stopwords). (3) Random swap randomly selects two words in a sentence and swaps their positions. (4) Random deletion deletes a word in a sentence with probability p.

**Figure 2:**
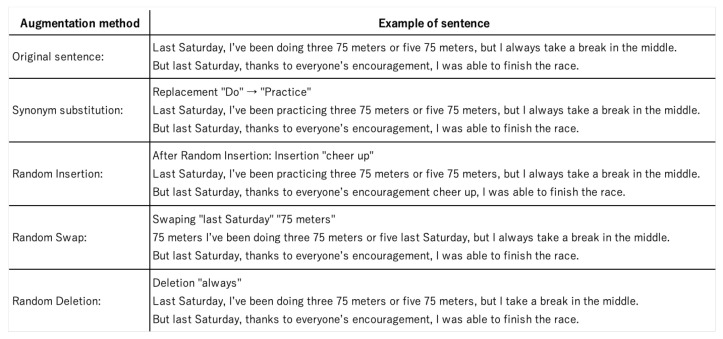
Original sentences in the dataset and sentences after using each of the EDA methods.

For the list of synonyms that needed to be replaced, the Japanese WordNet proposed by Isahara et al. was used for replacement [33]. The ratio of the text augmentation in each sample was denoted by a parameter α. Because a large value of α would reduce the accuracy, we set α to 0.05, as recommended by previous studies. Figure 2 illustrates an example sentence along with the four different types of augmentation results.

## 4. Evaluation

### 4.1. Evaluation Method

To evaluate the created model, all tasks in the dataset were used to perform automatic classification using BERT in the healthy older adults (MMSE 28 and above) and the group with MCI (MMSE 27 and below). There were 12 tasks in the dataset, involving 15 mildly cognitively impaired subjects and 45 healthy older subjects. The MCI group had 180 tasks and the normal older group had 540 tasks, so we augmented the MCI group 30 times and the normal older group 10 times (5400 samples each). A total of 10,800 data were divided into 10 parts for cross-validation, with 20% as test data and 10% as validation data, and the experiment was conducted.

### 4.2. Classification Using All Tasks

We tried all patterns from a learning rate of 2 $\times \;10^{-5}$ to 5 $\times \;10^{-5}$ and batch size of 1 to 32 and found that the learning rate: 2 $\times \;10^{-5}$ had the highest weighted average of F1-score with and without text augmentation.

**Figure 3:**
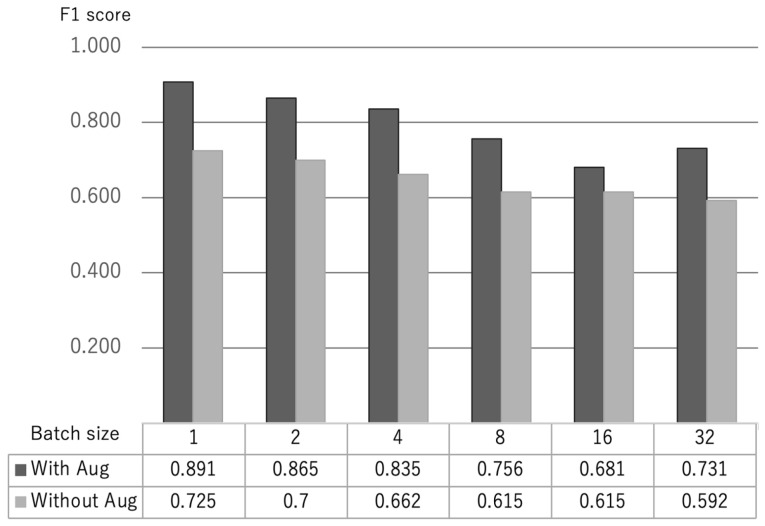
Weighted average of F1-scores per batch size with and without augmentation for learning rate 2 $\times \;10^{-5}$.

Without augmentation and learning rate: 2 $\times \;10^{-5}$, the weighted average of the F1-score was 0.725 for batch size 1, 0.700 for batch size 2, 0.662 for batch size 4, 0.615 for batch size 8, 0.615 for batch size 16, and 0.592 for batch size 32. With augmentation and learning rate: 2 $\times \;10^{-5}$, the weighted average of the F1-score was 0.891 for batch size 1, 0.865 for batch size 2, 0.835 for batch size 4, 0.756 for batch size 8, 0.681 for batch size 16, and 0.731 for batch size 32 (Figure 3).

As a result, the batch size of 1 and learning rate of 2 $\times \;10^{-5}$ had the highest weighted average of the F1-score, regardless of the presence or absence of augmentation (Figure 3). We obtained a weighted average F1-score = 0.725 without augmentation, and a weighted average F1-score = 0.891 with augmentation.

### 4.3. Classification Results for Each Task

A higher F1-score indicates that it is more accurate in saying whether someone was part of the MCI group or healthy control group. As a result, classified for each task: the F1-score for EP1a was 0.875 without augmentation and 0.957 with augmentation; the F1-score for EP1b was 0.786 without augmentation and 0.919 with augmentation. The F1-score of EP3a was 0.833 without augmentation and 0.804 with augmentation. The F1-score of EP4a was 0.750 without augmentation and 0.889 with augmentation; the F1-score of EP5a was 0.615 without augmentation and 0.908 with augmentation. The F1-score of EP6a was 0.833 without augmentation and 0.851 with augmentation; the F1-score of EP6b was 0.900 without augmentation and 0.905 with augmentation. The F1-score for EP7a was 0.667 without augmentation and 0.922 with augmentation. The F1-score for EP8a was 0.615 without augmentation and 0.923 with augmentation.

The F1-score for Picture was 0.909 without augmentation and 0.971 with augmentation, while the F1-score for Animation was 0.786 without augmentation and 0.939 with augmentation. The F1-score for Animation was 0.786 without augmentation and 0.939 with augmentation (Table 3).

## 5. Discussion

### 5.1. Effectiveness of Text Augmentation for Cognitive Function Classification

The improvement in classification accuracy with augmentation suggests that augmentation of the text of utterances is useful for detecting MCI levels. Although these results clearly indicate that arbitrary data can be increased, we believe that changes in classifiability for various factors such as gender and personality (e.g., Big Five personality traits) of the participant need to be verified in the future.

In addition, although ethical review is generally strict and there are many imbalanced datasets in medical fields, this study examined the effectiveness of text augmentation in the context of dementia. The results in this paper are likely to be applicable to the detection of other diseases, such as stroke and alcoholism, which can affect the language area of the brain, and may speed up medical intervention.

For future work, it is necessary to verify whether classification is possible not only for human-to-human conversation data but also for robot-to-human conversation data. If it is possible to classify the conversation data between a robot and a person, the home robotics with conversational function may allow us to monitor cognitive functions on a daily basis without going to a hospital or clinic. In addition, automatic speech recognition (ASR) may be used to extract textual information from speech data instead of manual transcription, which was performed in the dataset. However, errors such as incorrect word substitutions and unintended insertions may occur when using ASR. Therefore, the accuracy of ASR-based classification models may depend on the accuracy of ASR, which is outside the scope of this study. In recent years, tools that can automatically correct ASR and implement noise reduction have been improved, and it is expected that ASR-based language models will be applied in clinical settings in the future.

### 5.2. Parameters for Fine Tuning in BERT

For the parameters of fine tuning in BERT with the Japanese healthy and MCI older adults utterance dataset, the number of epochs was fixed to 4, since early stopping was applied, batch size was 1, 2, 4, 8, 16, 32, and learning rate (Adam) of 5 $\times \;10^{-5}$, 3 $\times \;10^{-5}$, 2 $\times \;10^{-5}$ were all tested. The results showed that the batch size of 1 and learning rate of 2 $\times \;10^{-5}$ gave the highest weighted average of the F1-score with and without augmentation. The authors of BERT recommend 16 and 32 for the batch size parameter, which is the recommended parameter when a single sentence contained 128 tokens. For data with long sentences, as in this case, it was found that lowering the batch size resulted in higher classification accuracy.

### 5.3. Individual Task-Based Analysis

In the contribution analysis of individual tasks, the Picture description task (0.971), EP1a: Recent sad event (0.957), and Animation description task (0.939) had the highest classification accuracy when there was augmentation, in that order. Both the Picture description task and the Animation description task required exposure to multiple events, which may have been a factor in their accuracy. For example, in the picture description task (PDT), a child trying to steal a cookie from a mother washing dishes in the kitchen is an important feature, whereas in the ADT, the interaction between two dogs and large and small unidentified life forms must be described in chronological order. Therefore, the descriptive terms that appeared in the healthy older participants were similar, whereas, conversely, the descriptions of events were sometimes inadequate in the texts of participants with impaired cognitive function. This difference may have increased the accuracy of the classification.

In contrast to the PDT and ADT, for which the correct answers are clear, the episodic task is a task to describe personal episodic memories, and the content of utterances differs from subject to subject. Therefore, the accuracy of classification was inferior, but more than six tasks achieved an accuracy of 0.9 or higher.

The task with higher F1-score weighted average among the episodic tasks was EP1a “which asked about a recent sad event”. In addition, EP7a. “People you admire” and EP8a “What you are currently passionate about” had a classification accuracy of 0.922 and 0.971, respectively, among the episodic tasks. This may be due to the relatively emotional expressions and lengthy explanations themselves. Conversely, EP3a, which had the lowest F1-score weighted average among the episodic tasks, asked about a recent angry event, suggesting that a social acceptance bias that subjects should not tell others about irritating events may have influenced their utterances [34].

In many previous studies, experiments were conducted using only the PDT. However, testing using only one task may have been influenced by the participant’s physical condition or mood at the time, and is not suitable for routine monitoring because it cannot be used repeatedly. However, this study suggests that adding other tasks (episode description task (EDT) or ADT) to the experiment may be able to detect dementia without decreasing the accuracy of classification.

## 6. Limitations

In this study, text augmentation of speech data from older patients with cognitive impairment was conducted. Since it can be difficult to obtain speech data for questions from patients with moderately or severely impaired cognitive impairment, further validation with a larger number of subjects is needed to determine what level of cognitive function is appropriate for this study.

## 7. Conclusions

In this paper, we evaluated the effectiveness of text augmentation on a dataset consisting of healthy Japanese-speaking older and MCI people using the Easy data augmentation method, utilizing a BERT model pre-trained for text analysis with a unique set of stopwords. The dataset contained 12 tasks for 60 older subjects (45 healthy older adults and 15 MCI elderly) and a total of 720 speech samples creating an imbalanced data set. For this dataset, we augmented the text data so that the data for the healthy older adults and the MCI patients were both 5400 samples each (a total data size of 10,800 samples). The results showed that the weighted average F1-score without augmentation was 0.725, and the weighted average F1-score with augmentation was 0.891, confirming the effectiveness of the augmentation.

We also examined which tasks contributed to the results. In the experiment with one episodic task at a time, the weighted average F1-score of the Picture description task (0.971) and the Animation description task (0.939) was higher when there was augmentation. The Picture description task and Animation description task both required exposure to multiple events, which may have been a factor. On the other hand, the episodic task is a task to describe personal episodic memories, and the content of utterances differs from subject to subject. Although the test using only one task is not suitable for daily monitoring because it cannot be used repeatedly, this study suggested the possibility of detecting dementia in daily conversation by adding other tasks. The results of this experiment could be used to detect other diseases that affect the language area of the brain, such as stroke and alcoholism, and may speed up medical intervention.

## Figures and Tables

**Table 1:** 10 items asked in the episode description task in the dataset.

No.	Content of Questions
(1)	1a: Recent sad event
(2)	1b: When did it happen?
(3)	2a: Recent event that made you feel anxious
(4)	3a: Recent event that made you angry
(5)	4a: Recent event that made you feel disgusted
(6)	5a: Recent surprising event
(7)	6a: Recent pleasant event
(8)	6b: When did it happen
(9)	7a: People you admire
(10)	8a: What you are currently passionate

**Table 2:** Number of participants, gender ratio, mean age, and mean Mini-Mental State Exam (MMSE) score of subjects.

	Healthy Older Group	MCI Older Group	Total
Number of participants	45	15	60
Gender ratio	M 23/F 22	M 8/F 7	M 31/F 29
Mean age	73.8 (±4.4)	73.5 (±5.5)	73.7 (±4.1)
Mean value of MMSE	29.3 (±0.7)	25.9 (±1.0)	28.4 (±1.6)

**Table 3:** Comparison of predicted correctness for each task in a model that classifies all tasks, with and without augmentation.

	Without Augmentation		With Augmentation	
Task	Rate of Correct Predictions	Number of Test Data	Rate of Correct Predictions	Number of Test Data
EP1a	0.875	8	0.957	188
EP1b	0.786	14	0.919	185
EP2a	0.778	9	0.894	180
EP3a	0.833	12	0.804	179
EP4a	0.750	16	0.889	199
EP5a	0.615	13	0.908	173
EP6a	0.833	12	0.851	174
EP6b	0.900	10	0.905	199
EP7a	0.667	12	0.922	179
EP8a	0.615	13	0.923	169
Picture	0.909	11	0.971	172
Animation	0.786	14	0.939	163
Average	0.779	12.0	0.907	180.0

## Data Availability

The data that support the findings of this study are available from the corresponding author upon reasonable request.
